# Liver X receptor and STAT1 cooperate downstream of Gas6/Mer to induce anti-inflammatory arginase 2 expression in macrophages

**DOI:** 10.1038/srep29673

**Published:** 2016-07-13

**Authors:** Si-Yoon Kim, Eun-Jin Lim, Young-So Yoon, Young-Ho Ahn, Eun-Mi Park, Hee-Sun Kim, Jihee Lee Kang

**Affiliations:** 1 Department of Physiology, School of Medicine, Ewha Womans University, Seoul 158-710, Korea; 2 Tissue Injury Defense Research Center, School of Medicine, Ewha Womans University, Seoul 158-710, Korea; 3 Department of Molecular Medicine, School of Medicine, Ewha Womans University, Seoul 158-710, Korea; 4 Department of Pharmacology, School of Medicine, Ewha Womans University, Seoul 158-710, Korea

## Abstract

Mer signaling increases the transcriptional activity of liver X receptor (LXR) to promote the resolution of acute sterile inflammation. Here, we aimed to understand the pathway downstream of Mer signaling after growth arrest-specific protein 6 (Gas6) treatment that leads to LXR expression and transcriptional activity in mouse bone-marrow derived macrophages (BMDM). Gas6-induced increases in LXRα and LXRβ and expression of their target genes were inhibited in BMDM from *STAT1*^−/−^ mice or by the STAT1-specific inhibitor fludarabine. Gas6-induced STAT1 phosphorylation, LXR activation, and LXR target gene expression were inhibited in BMDM from *Mer*^−/−^ mice or by inhibition of PI3K or Akt. Gas6-induced Akt phosphorylation was inhibited in BMDM from *STAT1*^−/−^ mice or in the presence of fludarabine. Gas6-induced LXR activity was enhanced through an interaction between LXRα and STAT1 on the DNA promoter of *Arg2*. Additionally, we found that Gas6 inhibited lipopolysaccharide (LPS)-induced nitrite production in a STAT1 and LXR pathway-dependent manner in BMDM. Additionally, Mer-neutralizing antibody reduced LXR and Arg2 expression in lung tissue and enhanced NO production in bronchoalveolar lavage fluid in LPS-induced acute lung injury. Our data suggest the possibility that the Gas6-Mer-PI3K/Akt-STAT1-LXR-Arg2 pathway plays an essential role for resolving inflammatory response in acute lung injury.

Liver X receptors (LXR) were originally identified as ligand-dependent transcriptional activators belonging to a nuclear receptor superfamily^1^,^2^. The LXR subfamily consists of two isoforms, LXRα and LXRβ, that are highly related and share ~78% amino acid sequence identity in both their DNA and ligand-binding domains^3^. After binding oxysterol ligands, LXRs are activated and induce target genes involved in cholesterol and lipid metabolism, including ATP-binding cassette, sub-family A (*ABCA1*), ATP-binding cassette, sub-family G (*ABCG1*), apolipoprotein E (*ApoE*), and phospholipid transfer protein (*PLTP*)^4^,^5^,^6^,^7^. Recently, LXRs have emerged as important regulators of inflammatory gene expression, innate immunity, and autoimmune diseases^8^,^9^,^10^,^11^. For example, genes such as inducible nitric oxide synthase (*iNOS*), interleukin-6 (*IL-6*), cyclooxygenase-2 (*COX-2*), matrix metallopeptidase-9 (*MMP-9*), tumor necrosis factor α (*TNF-α*), and *IL-1β* are induced in macrophages exposed to bacterial pathogens, Toll-like receptor (TLR) stimulants, or proinflammatory cytokines, but can be repressed by LXR activation, which antagonizes transcription factor activity^8^,^9^ or inhibits the release of corepressor complexes from target gene promoters^10^. Moreover, LXR has been demonstrated to up-regulate the expression of genes involved in immune and inflammatory responses, such as apoptotic inhibitor of macrophages (*AIM*), arginase 2 (*Arg2*), and vascular endothelial growth factor (*VEGF*)^11^. Gene expression and promoter analysis confirmed that these genes are direct targets of LXR/RXR heterodimers and their promoters contain a functional liver X receptor element (LXRE). The expression and activity of LXRs in various cell types are strictly regulated. However, it is considered that expression of LXRs and the presence of appropriate ligands are usually not to elicit optimal or maximal responses^12^. Further transcriptional mechanisms confer to enhance or limit responsiveness leading to cell type- or condition-specific gene expression pattern^9^,^12^.

Mer is a cell membrane-bound receptor tyrosine kinase that, together with Axl and Tyro3, constitutes the TAM receptor family^13^. Growth arrest-specific protein 6 (Gas6) and anticoagulant protein S are important biological ligands for the TAM receptors^14^,^15^. Ligand interaction with TAM receptors leads to receptor phosphorylation and activation of downstream signaling pathways that affect numerous functions, including cell survival, thrombosis, proliferation, cell migration, and phagocytosis of apoptotic cells. We recently demonstrated that endogenous Mer activation plays a critical role in the resolution of acute sterile inflammation^16^. Importantly, we elucidated a previously uncharacterized pathway that involves Mer signaling in peritoneal macrophages, spleen, and lung that leads to a progressive increase in LXR mRNA and protein abundance and activation over the course of acute inflammation. However, the post-receptor signaling pathway between Mer and LXR expression and activation has not been well characterized.

Here, we aimed to understand the pathways downstream of Gas6/Mer signaling that lead to LXR expression and transcriptional activity in mouse bone-marrow derived macrophages (BMDM). We focused on the role of the STAT1 transcription factor, which acts as an enhancer of LXR expression and activation. In addition, we propose a mechanism by which STAT1 enhances LXRα binding to the promoter of an anti-inflammatory target gene, *Arg2*, which leads to increased LXRα responsiveness and reciprocal NO production. We further show that GAS6/Mer signaling inhibits chemotaxis and enhances IL-10 secretion via LXR pathway. Moreover, we provide *in vivo* evidence using Mer-neutralizing antibody that Gas6/Mer signaling modulates NO production through activation of LXR/Arg2 pathway in LPS-induced acute lung injury, which in part promotes the resolution of acute lung injury^17^.

## Results

### Gas6/Mer signaling enhances LXRα and LXRβ activity in mouse BMDM

**Figure 1:**
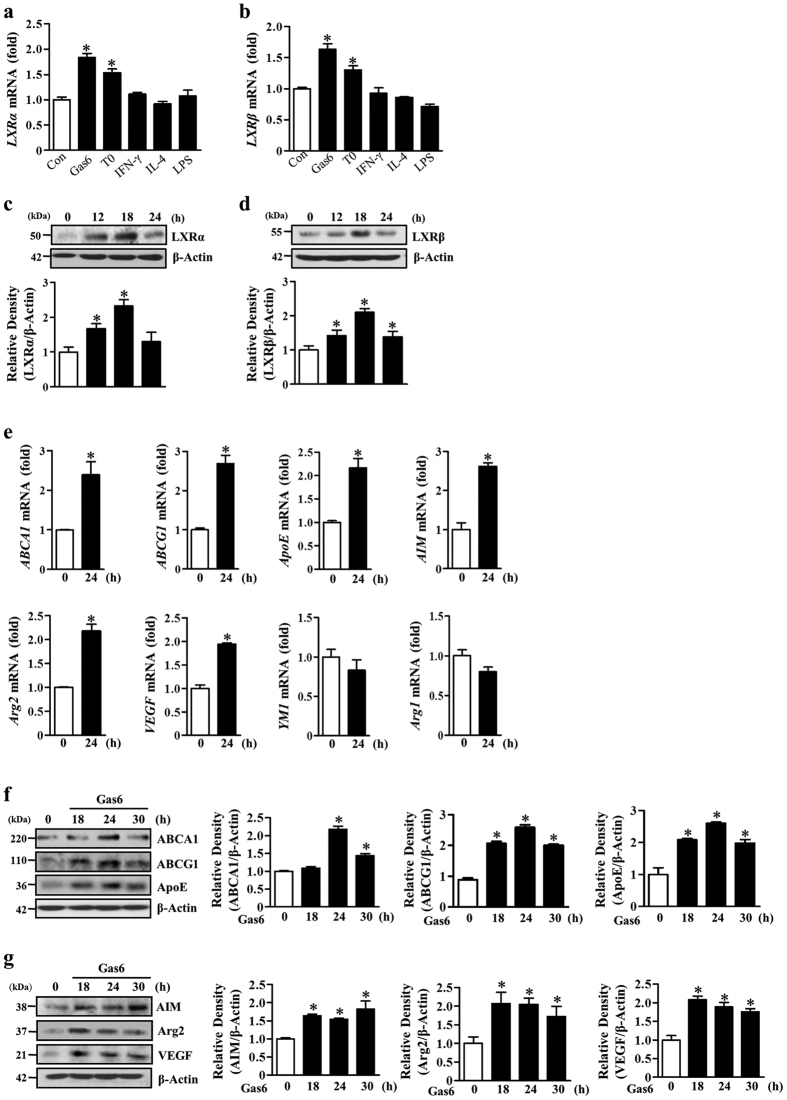
Gas6 treatment enhances expression of LXRα and LXRβ and their target genes in BMDM. Mouse BMDM were stimulated with 400 ng/ml Gas6, 1 μM T0901317, 10 ng/ml interferon (IFN)-γ, 10 ng/ml IL-4, or 100 ng/ml LPS for 4 h (**a,b**) or 400 ng/ml Gas6 for the indicated times (**c**–**g**). (**a,b,e**) The amounts of the *LXRα, LXRβ, ABCA1, ABCG1, ApoE, AIM, Arg2, VEGF, YM1*, and *Arg1* mRNAs were analyzed by real-time PCR and normalized to that of *Hprt* mRNA. (**c,d,f,g**) The relative abundances of LXRα, LXRβ, ABCA1, ABCG1, ApoE, Aim, Arg2, and VEGF proteins were determined by Western blotting analysis. The relative densitometric intensity was determined for each band and normalized to β-actin. Data in all bar graphs are means ± SEM of three independent experiments. *P < 0.05 compared with control.

We previously showed that Gas6/Mer engagement induces LXR expression and transcriptional activity in RAW 264.7 macrophages^16^. In the present study, to confirm the impact of Gas6/Mer signaling on LXR activity in mouse BMDM, we measured mRNA and protein abundance of LXR and LXR target gene expression for 24 h after Gas6 treatment. Similar to previous findings, the induction of *LXRα* and *LXRβ* in BMDM was rapid and peaked at 2 h. We found sustained *LXRα* expression up to 4 h after exposure to Gas6, which gradually declined until 12 h and then slightly increased at 24 h after Gas6 treatment (Supplementary Fig. S1a). A similar time course of *LXRβ* expression was also found (Supplementary Fig. S1b). In comparison, Gas6 did not induce *RXR* mRNA expression up to 8 h (Supplementary Fig. S1c). Interestingly, the abundance of *LXRα* and *LXRβ* mRNA after Gas6 treatment was greater than after treatment with 1 μM T0901317, an LXR agonist (Fig. 1a,b). However, other immunomodulators, such as interferon (IFN)-γ (10 ng/ml), IL-4 (10 ng/ml), and lipopolysaccharide (LPS, 100 ng/ml), did not stimulate an induction of *LXRα* and *LXRβ* mRNA in BMDM. In addition, LXRα and LXRβ protein abundance in BMDM was continuously enhanced up to 18 h after exposure to Gas6 (Fig. 1c,d).

Concomitant with the increase in LXR, several of its well-established targets, such as *ABCA1, ABCG1*, and *ApoE,* which are involved in lipid and cholesterol metabolism, were substantially increased at the mRNA and protein level in BMDM following Gas6 exposure, indicating increased functional transcriptional activity of LXR (Fig. 1e,f). In addition, the induction of other direct LXR target genes that are involved in immune and inflammatory responses, such as *AIM, Arg2*, and *VEGF*, also occurred in BMDM after Gas6 treatment (Fig. 1e,g). However, the induction of other alternative activation markers, but not direct target genes of LXR, such as chitinase 3-like 1 (*YM1*) and *Arg1*, which are highly induced by PPARγ-dependent mechanisms, was not observed at the mRNA level (Fig. 1e).

**Figure 2:**
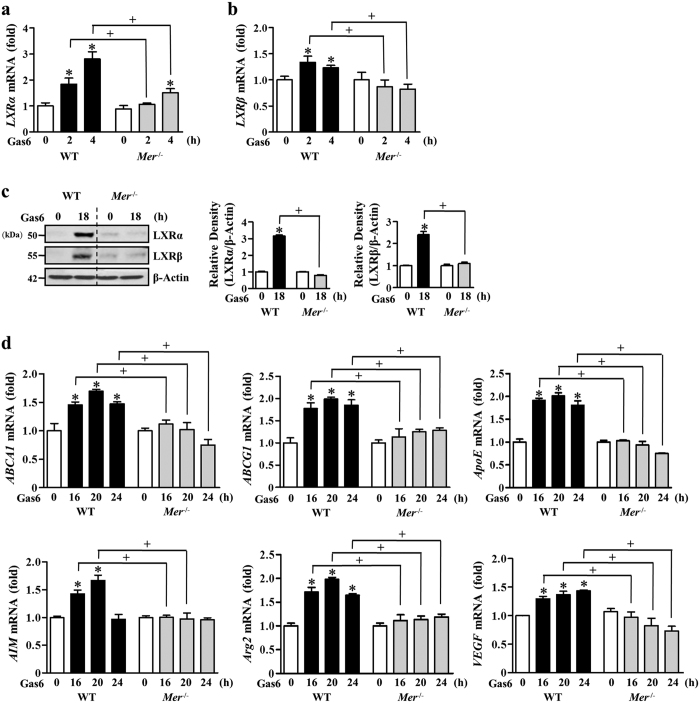
Gas6-induced LXRα and LXRβ transcriptional response is decreased in *Mer*- deficient BMDM. Mouse BMDM from wild-type or *Mer*^−/−^ mice were stimulated with 400 ng/ml Gas6 for the indicated times (**a**–**d**). (**a,b,d**) The amounts of the *LXRα, LXRβ, ABCA1, ABCG1, ApoE, AIM, Arg2*, and *VEGF* mRNAs were analyzed by real-time PCR and normalized to that of *Hprt* mRNA. (**c**) The relative abundances of LXRα and LXRβ proteins were determined by Western blotting analysis. The relative densitometric intensity was determined for each band and normalized to β-actin. Data in all bar graphs are means ± SEM of three independent experiments. *P < 0.05 compared with control; ^+^*P *< 0.05 as indicated.

In addition, we confirmed the proposal that Mer is engaged in mediating the increased abundance of LXR and activation in BMDM by using *Mer*^−/−^ mice. Gas6-induced LXRα and LXRβ mRNA and protein expression was inhibited in BMDM from *Mer*^−/−^ mice (Fig. 2a–c). Concomitantly, mRNA abundance of their target genes, such as *ABCA1, ABCG1, ApoE, AIM, Arg2*, and *VEGF* in BMDM from *Mer*^−/−^ mice was substantially decreased compared with those of WT mice (Fig. 2d). These data suggest that Mer signaling in BMDM requires Gas6-induced LXRα and LXRβ as well as transactivation of their target genes, including anti-inflammatory molecules or alternative activation markers of macrophages.

### STAT1 is required for Gas6/Mer signaling-induced LXR expression and activation in BMDM

**Figure 3:**
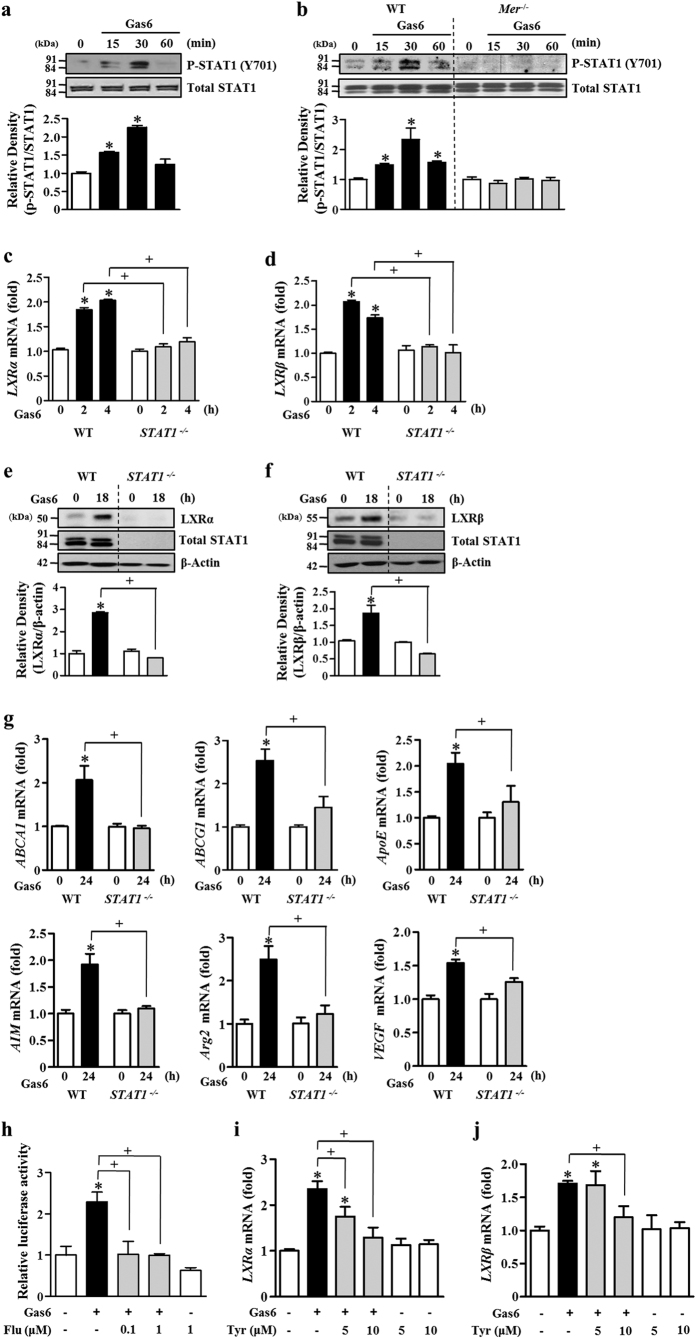
STAT1 activation mediates Gas6-induced increases in LXRα and LXRβ as well as *ABCA1, ABCG1, ApoE, AIM, Arg2*, and *VEGF* expression. BMDM from C57BL/6 mice (**a**) or from *Mer*^−/−^ and WT mice (**b**) were stimulated with 400 ng/ml Gas6 for the indicated times. BMDM from WT or *STAT1*^−/−^ mice were stimulated with 400 ng/ml Gas6 for the indicated times (**c**–**g**). (**h**) RAW 264.7 cells were co-transfected with an LXRE-driven luciferase reporter vector and a Renilla control plasmid. After 24 h, cells were treated with 400 ng/ml Gas6 in the absence or presence of fludarabine at the indicated concentrations. LXR activity was then measured by the dual luciferase assay. (**i,j**) BMDM from C57BL/6 mice were pretreated with vehicle or the indicated concentrations of the JAK2 inhibitor tyrphostin AG490 (Tyr) for 1 h before being treated with 400 ng/ml Gas6. (**a,b,e,f**) The relative abundances of total STAT1, phosphorylated STAT1 (Y701), LXRα, and LXRβ proteins were determined by Western blotting analysis. The relative densitometric intensity was determined for each band and normalized to the indicated proteins. (**c,d,g,i,j**) The amounts of the *LXRα, LXRβ, ABCA1, ABCG1, ApoE, AIM, Arg2*, and *VEGF* mRNAs were analyzed by real-time PCR and normalized to that of *Hprt* mRNA. Data in all bar graphs are means ± SEM of three independent experiments. *P < 0.05 compared with control; ^+^*P *< 0.05 as indicated.

Previously, the transduction of the anti-inflammatory effects of TAM receptors appeared to involve the JAK/STAT1 pathway in dendritic cells^18^. Following tyrosine phosphorylation, the STAT1 homodimer is generated and translocates to the nucleus. Thus, we focused on determining whether this pathway impacts Gas6/Mer signaling-induced LXR expression and activity. First, we examined the time course of activation of STAT1 in BMDM after 400 ng/ml Gas6 stimulation. STAT1 phosphorylation (tyrosine 701) was rapidly enhanced and peaked at 30 min (Fig. 3a). STAT1 tyrosine phosphorylation declined to the control level at 60 min after Gas6 treatment. In contrast, serine phosphorylation of STAT1 did not occur in BMDM treated with Gas6 (Supplementary Fig. S2). Notably, STAT1 tyrosine phosphorylation was not observed in Mer-deficient BMDM in response to Gas6 (Fig. 3b). Furthermore, treatment with the LXR agonist T0901317 (1 μM) did not enhance STAT1 tyrosine phosphorylation in BMDM (Supplementary Fig. S3).

A recent study showed that STAT3 is activated in a Gas6/Mer-dependent manner in mouse bone marrow-derived dendritic cells (BMDC)^19^. Thus, we examined whether Gas6/Mer signaling induces STAT3 phosphorylation in BMDM. Unlike to STAT1 activation, STAT3 phosphorylation was not induced until 60 min in BMDM after 400 ng/ml Gas6 treatment (Supplementary Fig. S4).

To verify the involvement of STAT1 activation in Gas6-induced LXR expression and functional activation, BMDM from *STAT1*^−/−^ and WT mice were treated with Gas6. mRNA and protein induction of LXRα and LXRβ was completely suppressed in genetically *STAT1*-deficient BMDM but enhanced in WT BMDM following Gas6 treatment (Fig. 3c–f). Moreover, Gas6-induced mRNA expression of LXR target genes, such as *ABCA1, ABCG1, ApoE, AIM, Arg2*, and *VEGF*, was inhibited in *STAT1*-deficient BMDM (Fig. 3g). Similarly, the STAT1 specific inhibitor fludarabine (1 μM) inhibited Gas6-mediated induction of LXRα and LXRβ mRNA and protein in a dose-dependent manner (Supplementary Fig. S5a–d). LXRα and LXRβ target gene expression was also partially inhibited by treatment with 1 μM fludarabine (Supplementary Fig. S5e). Moreover, inhibition of STAT1 signaling by fludarabine prevented Gas6-induced increases in LXRE activity in RAW 264.7 cells (Fig. 3h).

Next, to further probe the involvement of the JAK/STAT1 pathway in Gas6-mediated LXR activation, BMDM were pretreated with the JAK2 inhibitor tyrphostin AG490 (5 and 10 μM) or the JAK3 inhibitor WHI-P131 (5 and 10 μM) for 1 h prior to treatment with Gas6. The JAK2 inhibitor (Fig. 3i,j), but not the JAK3 inhibitor (Supplementary Fig. S6a,b), significantly suppressed Gas6-mediated induction of LXRα and LXRβ mRNA and protein. Taken together, these data suggest that the JAK2/STAT1 pathway is required, at least in part, for the induction of LXRα and LXRβ expression and for LXR target gene expression.

### Gas6-induced PI3K/protein kinase B (Akt) and STAT1 pathways positively cross-talk in mouse BMDM

Our previous study demonstrated that Akt activation is involved in Gas6-induced LXR mRNA and protein expression in RAW 264.7 cells^16^. Thus, in the present study, we further examined whether STAT1 signaling interacts with other known downstream pathways, such as PI3K/Akt. First, we examined the time course of activation of Akt in BMDM after Gas6 stimulation. Gas6 induced Akt phosphorylation with a peak phosphorylation at 15 min, which then gradually declined until 90 min (Supplementary Fig. S7a). To examine the involvement of the PI3K/Akt pathway in LXR induction after Gas6 stimulation, mouse BMDM were pretreated with PI3K inhibitors, such as LY294002 (10 μM) and wortmannin (100 nM), or the Akt inhibitor MK2206 (1 μM), for 1 h prior to stimulation with Gas6. All of these drugs inhibited Gas6-mediated induction of LXRα and LXRβ mRNA and protein (Supplementary Fig. S7b–g). Moreover, MK2206 inhibited protein expression of LXR target genes, such as AIM, Arg2 and VEGF (Supplementary Fig. S7h–j). These data confirmed that the PI3K/Akt pathway is also involved in LXRα and LXRβ expression and activation in BMDM treated with Gas6.

**Figure 4:**
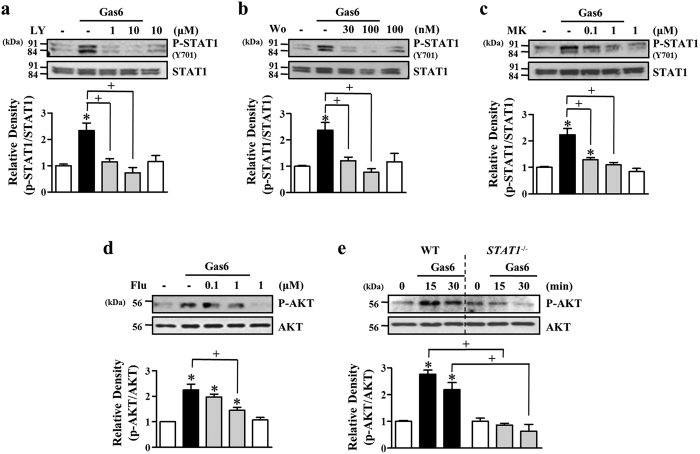
PI3K/Akt and STAT1 pathways interact positively upon stimulation with Gas6. BMDM from C57BL/6 mice were stimulated with 400 ng/ml Gas6 for 30 min. (**a–d**) BMDM were pretreated with vehicle or the PI3K-specific inhibitor LY294002 (LY), wortmannin (Wo), the Akt inhibitor MK2206 (MK), or the STAT1-specific inhibitor fludarabine (Flu) at the indicated concentrations before 400 ng/ml Gas6 treatment. (**e**) BMDM from WT or *STAT1*^−/−^ mice were stimulated with 400 ng/ml Gas6 for the indicated times. The relative abundances of total Akt, phosphorylated Akt, total STAT1, and phosphorylated STAT1 were determined by Western blotting analysis. The relative densitometric intensity was determined for each band and normalized to the indicated proteins. Data in all bar graphs are means ± SEM of three independent experiments. *P < 0.05 compared with control; ^+^*P *< 0.05 as indicated.

As described above, activation of the STAT1 and PI3K/Akt pathways is required for Gas6-induced LXRα and LXRβ mRNA and protein production in BMDM. Thus, we investigated how PI3K/Akt and STAT1 pathways cross-talk upon stimulation of BMDM with Gas6. First, we examined whether the PI3K/Akt pathway acts as an upstream regulator of the STAT1 pathway. PI3K inhibitors, such as LY (1 and10 μM) and wortmannin (30 and 100 nM), and the Akt inhibitor MK (0.1 and 1 μM) suppressed Gas6-induced tyrosine phosphorylation of STAT1 (Y701) (Fig. 4a–c). Next, we examined the action of STAT1 on Akt activation in Gas6-stimulated BMDM. Gas6-induced phosphorylation of Akt was reduced by pretreatment with the STAT1 inhibitor and in *STAT1*-deficient BMDM (Fig. 4d,e). These data suggest that the PI3K/Akt and STAT1 pathways positively cross-talk upon stimulation with Gas6 in mouse BMDM, leading to LXR expression and activation.

### Gas6-induced STAT1 and Akt phosphorylation is Mer independent in mouse BMDC

In addition to BMDM, we also examined whether Gas6/Mer signaling engagement induces STAT1 and Akt phosphorylation and LXRα expression in BMDC from WT and Mer^−/−^ mice. Phosphorylation of STAT1 and Akt was induced by Gas6 treatment in both WT and Mer^−/−^ BMDC, indicating the increase in phosphorylation of STAT1 and Akt was Mer independent (Supplementary Fig. S8a,b). Unlike to BMDM, Gas6 stimulation did not enhance LXRα and LXRβ gene expression in BMDC from WT mice (Supplementary Fig. S9a,b).

### STAT1 facilitates LXRα activity on the *Arg2* gene promoter after stimulation with Gas6

**Figure 5:**
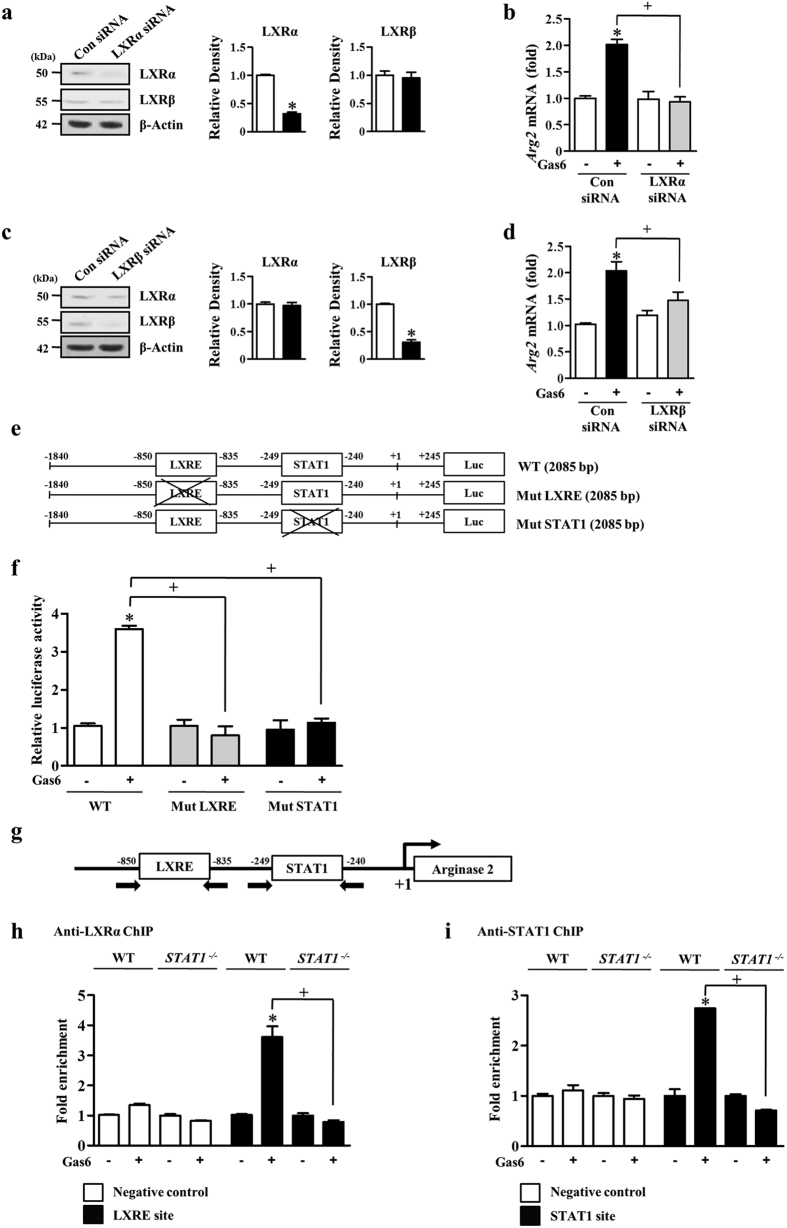
Gas6 increases *Arg2* promoter activity through LXRE and STAT1 binding. (**a–d**) RAW 264.7 cells were transfected with *LXRα*- or *LXRβ*-specific siRNA or negative control siRNA. After 24 h, the cells were treated with 400 ng/ml Gas6 for. (**a,c**) The relative abundances of LXRα and LXRβ proteins were determined by Western blotting analysis. Densitometric analysis of the relative abundances of the indicated proteins. (**b,d**) The relative abundance of *Arg2* mRNA was determined by real-time PCR. (**e,f)** Site-directed mutagenesis of the *Arg2* promoter. (**e**) Diagrams of the *Arg2* promoter constructs (−1840 to +245; 2085 bp) with transcription factor binding sites represented by white boxes (LXRE site: −1108 to −1094; STAT1 site: −507 to −499; Luc, firefly luciferase). The crossed boxes denote the mutated sites. (**f**) Promoter reporter constructs were transfected into RAW 264.7 cells followed by treatment with or without Gas 6 (400 ng/ml) for 24 h. For each sample, firefly luciferase activity was normalized to Renilla luciferase activity. (**g**) Diagram of the *Arg2* promoter region with putative transcription factor binding sites (LXRE: −1108 to −1094; STAT1: −507 to −499). Arrows indicate PCR primer binding sites. (**h,i**) Chromatin immunoprecipitation (ChIP) assays using anti-LXRα (**h**) and anti-STAT1 (**i**) antibodies were performed in BMDM from WT and *STAT1*^−/−^ mice in the presence or absence of 400 ng/ml Gas6. A genomic locus around 4 kb upstream from the transcription start site of *Arg2* was used to measure background binding. Data in all bar graphs are means ± SEM of three independent experiments. *P < 0.05 compared with control; ^+^*P *< 0.05 as indicated.

We chose an LXR anti-inflammatory target gene, Arg2^20^, to study the LXR response at the promoter level. Previous studies have shown that the LXRE consensus binding site located upstream of the *Arg2* transcription initiation site is required for *Arg2* promoter activity in RAW 264.7 cells^20^. In the present study, RAW 264.7 cells were transfected with *LXRα*- or *LXRβ*-specific siRNA or with a negative control siRNA and then were cultured for 48 h. The abundances of LXRα or LXRβ protein were specifically decreased by ~70% in cells transfected with *LXRα*- or *LXRβ*-specific siRNAs compared to those in RAW 264.7 cells transfected with negative control siRNA (Fig. 5a,c). We found that the reduction of *Arg2* mRNA by *LXRα* siRNA upon stimulation with Gas6 was more prominent than that caused by *LXRβ* siRNA (Fig. 5b,d). Thus, we monitored the LXRα response on *Arg2* promoter activity in RAW 264.7 cells. We used a luciferase reporter construct containing the promoter region of mouse *Arg2* from −1840 to +245 base pairs (bp) relative to the transcription initiation site (+1). In addition to the LXRα sites (−1108 to −1094), we also identified a potential STAT1 site (−507 to −499) by using the JASPAR database at http://jaspar.genereg.net/ (Fig. 5e). To determine the requirement of specific regulatory elements for Gas6-induced *Arg2* promoter activity, we mutated the STAT1 and LXRE consensus sequences within the *Arg1* promoter region. As shown in Fig. 5f, mutation of the LXRE site abolished Gas6-induced promoter activity, indicating that the LXRE site contributes to Gas6-mediated induction of the *Arg2* gene. Interestingly, mutation of the STAT1 binding site also completely blocked Gas6-induced promoter activity. These data strongly suggest that STAT1 could be one of the transcription factors that enhances *Arg2* gene induction associated with LXRα-binding activity to LXRE. This implication raised the possibility of physical interaction between the two transcription factors, LXRα and STAT1.

To confirm the binding of LXRα and STAT1 to the *Arg2* promoter, we performed ChIP analysis using anti-LXRα or anti-STAT1 antibodies in BMDM with or without Gas6 (Fig. 5g). LXRα and STAT1 preferentially bound to the corresponding sites on the *Arg2* promoter in Gas6-stimulated BMDM but not unstimulated cells (Fig. 5h,i). Importantly, the recruitment of LXRα to the *Arg2* promoter by Gas6 was markedly reduced in BMDM from *STAT1*^−/−^ mice, indicating that LXRα and STAT1 might physically or functionally interact *in vivo*.

### STAT1 signaling-dependent LXR activation inhibits nitric oxide (NO) production in BMDM

**Figure 6:**
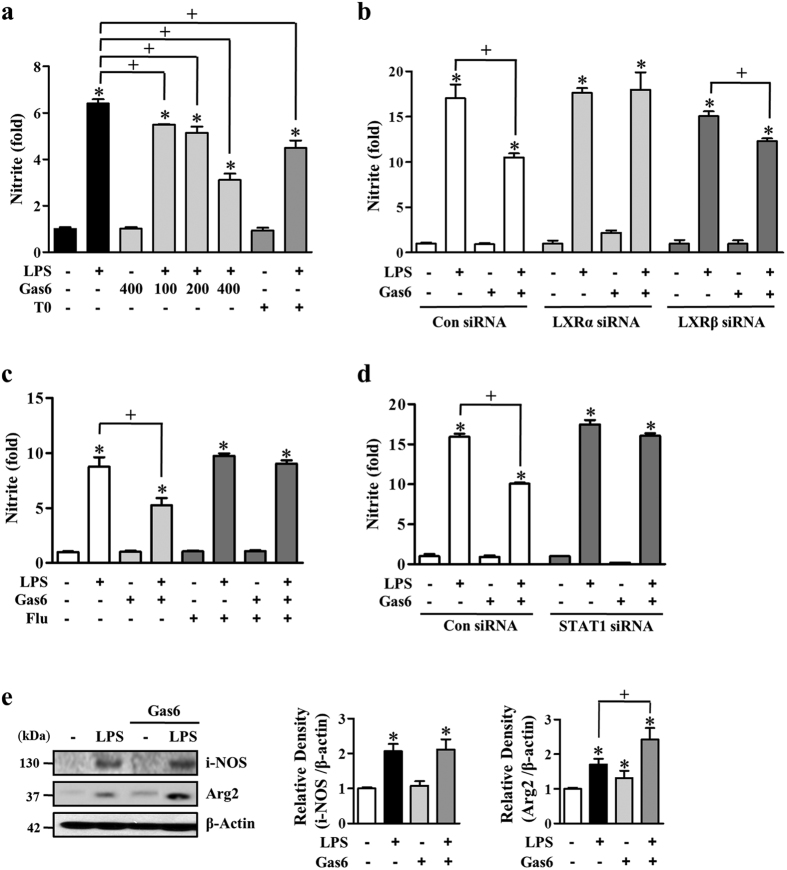
Gas6 inhibits nitrite production in BMDM via STAT1-dependent LXR activation. (**a**) Mouse BMDM were stimulated with 100–400 ng/ml Gas6 or 1 μM T0901317 for 24 h before 100 ng/ml LPS treatment. (**b,d**) Mouse BMDM were transfected with either control siRNA, *LXRα*-, *LXRβ*-, or *STAT1*-specific siRNA. After 18 h, the cells were treated with Gas6 before LPS treatment, as indicated. (**c**) Mouse BMDM were pretreated with vehicle or 1 μM fludarabine for 1 h before Gas6 treatment (400 ng/ml). (**a**–**d**) At 24 h after LPS treatment, nitrite accumulation in the culture media was measured using Griess reagent. (**e**) The relative abundances of i-NOS and Arg2 proteins were determined by Western blotting analysis. The relative densitometric intensity was determined for each band and normalized to β-actin. Data in all bar graphs are means ± SEM of three independent experiments. *P < 0.05 compared with control; ^+^*P *< 0.05 as indicated.

Previously, it was demonstrated that LXR activation inhibits NO production in LXR- or Arg2-overexpressing macrophages^20^. In the present study, we investigated whether Gas6-induced LXR activation via STAT1 inhibits NO production in BMDM. First, we performed a nitrite assay in 100 ng/ml LPS-stimulated BMDM in the presence or absence of 100–400 ng/ml Gas6 or 1 μM of the LXR agonist T0901317. Gas6 inhibited LPS-induced nitrite production in a concentration-dependent manner, with 60% inhibition at 400 ng/ml (Fig. 6a). In comparison, T0901317 also inhibited LPS-induced nitrite production with 35% inhibition. Next, to verify that LXR functions downstream of Gas6 signal in nitric oxide production, mouse BMDM were transfected with *LXR* or negative control siRNA and cultured for 66 h. The abundance of LXRα or LXRβ was specifically decreased by ~70% in cells transfected with *LXRα*- or *LXRβ*-specific siRNA compared to cells transfected with the negative control siRNA (Supplementary Fig. S10a,b). Gas6 was unable to inhibit the LPS-induced nitrite production in BMDM in which the LXR isoforms were silenced individually, while nitrite production in negative control siRNA-transfected cells was inhibited by Gas6 (Fig. 6b). In addition, pharmacological STAT1 inhibition reversed the inhibitory effect of Gas6 on LPS-induced nitrite production in BMDM (Fig. 6c). Similarly, knockdown of STAT1 protein by transfection of BMDM with a *STAT1*-specific siRNA (~65% reduction, Supplementary Fig. S10c) reversed the Gas6-induced reduction of nitrite production (Fig. 6d). We further established that the reduced NO production in BMDM pretreated with Gas6 was not a consequence of decreased iNOS but increased Arg2 protein expression (Fig. 6e). The abundance of iNOS protein was equivalently induced by LPS in BMDM in the presence or absence of Gas6.

### Gas6 suppresses macrophage chemotaxis in BMDM and enhances secretion of IL-10

**Figure 7:**
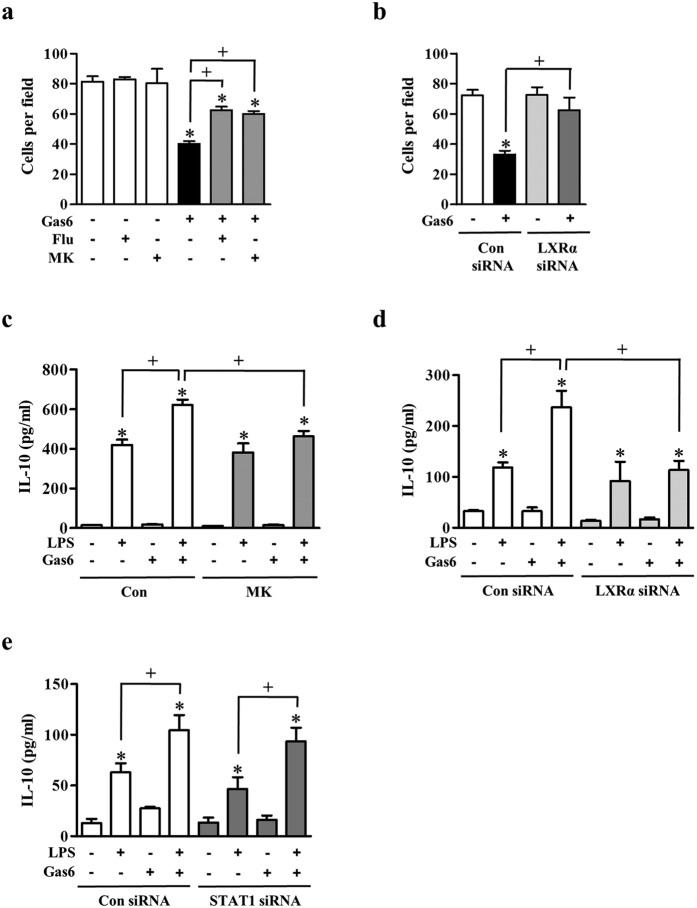
Gas6 inhibits macrophage chemotaxis and enhances secretion of IL-10. (**a**) The number of BMDM migrated across the transwell membrane after 18 h treatment with Gas6 in the absence or presence of fludarabine (1 μM) or MK2206 (1 μM). (**b**) After BMDM was transfected with either control siRNA or *LXRα*-specific siRNA for 18 h, the migrated cell number across the transwell membrane after 18 h treatment with Gas6. (**a**,**b**) Chemotaxis was quantified by counting BMDM on the undersides of the membrane. (**c–e**) BMDM were stimulated with 400 ng/ml Gas6 for 24 h before 100 ng/ml LPS treatment. (**c**) BMDM were pretreated with vehicle or 1 μM MK2206 (MK) for 1 h before Gas6 treatment. (**d**,**e**) Mouse BMDM were transfected with either control siRNA, *LXRα*-, or *STAT1*-specific siRNA. After 18 h, the cells were treated with Gas6 before LPS treatment, as indicated. (**c**–**e**) At 24 h after LPS treatment, IL-10 secretion in the culture media was measured by ELISA. Data in all bar graphs are means ± SEM of three independent experiments. *P < 0.05 compared with control; ^+^*P *< 0.05 as indicated.

We further studied to determine whether the Gas6/Mer/Akt/STAT1/LXR pathway affects macrophage chemotaxis and pro-resolving cytokine secretion. We examined the effect of this pathway on macrophage chemotaxis by counting the number of BMDM crossing a transwell membrane in the presence of 400 ng/ml Gas6. Gas6 inhibited BMDM migration toward chemoattractants in the bottom well compared with the control group (50% inhibition) (Fig. 7a). However, fludarabine or MK2206 reversed this suppressive effect of Gas6 on chemotaxis. Similarly, knockdown of LXRα protein by transfection of BMDM with a LXRα-specific siRNA reversed the Gas6-induced inhibition of chemotaxis (Fig. 7b).

Gas6 significantly reduced LPS-induced production of TNF-α and tended to increase LPS-induced IL-10 production via Mer^21^,^22^. Here, we examined the effect of Gas6-induced Akt-STAT1-LXR pathway on macrophage function with secretion of a pro-resolving cytokine IL-10, which represents a central functional property of M2 macrophages^23^. Gas6 further enhanced LPS-induced IL-10 production by ~50% increase in BMDM (Fig. 7c). This enhancement was inhibited by pretreatment with MK2206, or transfection with LXRα specific siRNA (Fig. 7c,d) However, transfection of BMDM with a *STAT1*-specific siRNA did not significantly inhibit the Gas6-induced IL-10 production (Fig. 7e).

### Inhibiting Mer signaling suppresses LXR and Arg2 expression and enhances NO production in LPS-induced acute lung injury

**Figure 8:**
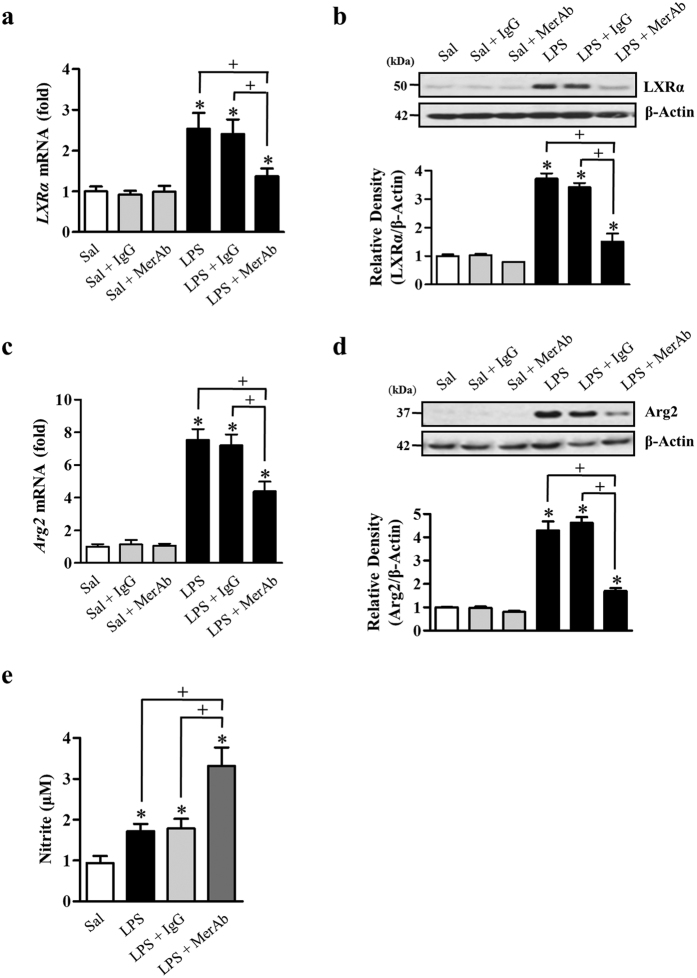
The anti-Mer antibody reduces mRNA and protein abundances of LXRα and Arg2 and enhances NO production in LPS-induced acute lung inflammation. Where indicated, mice were treated i.t. with LPS or given 2 mg/kg anti-Mer or IgG antibody i.v. 1 h before LPS treatment. Animals were sacrificed at 24 h post-LPS treatment. (**a,c**) Expression of mRNA in the lung was analyzed by real-time PCR and normalized to that of *Hprt* mRNA. (**b,d**) Western blots probed with anti-LXRα, or anti-Arg2 Ab were employed in the lung homogenates. The relative densitometric intensity was determined for each band and normalized to α-tubulin. (**e**) Nitrite accumulation in the BAL fluid was measured using Griess reagent. The values represent the mean ± SEM of five mice in each group. *P < 0.05 compared with saline control, ^+^P < comparing the LPS + anti-Mer group versus the LPS-treated group or the LPS + IgG group at a given time.

In our previous work, we found that the inhibition of Mer signaling during acute pulmonary inflammation with an anti-Mer neutralizing antibody led to hyper-responsive TLR4 activation by LPS^17^. Moreover, we found that pretreatment with anti-Mer antibody significantly reduced Mer activation, Akt phosphorylation, and STAT1 activation during LPS-induced acute lung inflammation, indicating involvement of Akt and STAT1 as the downstream molecules of Mer activation in anti-inflammatory response in acute lung injury. In the present study, we further examined whether treatment with anti-Mer antibody changes in mRNA and protein abundances of LXRα and Arg2 in lung tissue at 24 h after intratracheal instillation of LPS. The mRNA and protein abundances of LXRα and Arg2 in lung tissue from LPS-treated mice were reduced by anti-Mer antibody treatment (Fig. 8a–d). In contrast, NO production in bronchoalveolar lavage (BAL) fluid was further enhanced by anti-Mer Ab treatment at 24 h after LPS treatment (Fig. 8e). However, pretreatment with IgG did not show these changes.

## Discussion

We previously reported that Gas6 enhances the abundance of LXRα and LXRβ and their target genes, which are involved in cholesterol and lipid metabolism, in RAW 264.7 cells^16^. Here, we confirmed that Mer signaling is involved in Gas6-induced LXRα and LXRβ expression and activation in mouse BMDM, as LXRα and LXRβ mRNA and protein were reduced in BMDM from *Mer*^−/−^ mice. In addition to LXR target genes involved in cholesterol and lipid metabolism, including *ABCA1, ABCG1*, and *ApoE,* the mRNA expression of *AIM, Arg2*, and *VEGF*, which are known to mediate immune and inflammatory responses, was also reduced in Mer-deficient BMDM. These data show for the first time that Gas6/Mer signaling enhances LXRα and LXRβ expression and the transactivation of anti-inflammatory molecules in BMDM.

In the present study, we focused on identifying which downstream signaling pathway is required for the induction of LXR expression and transcriptional response upon Gas6 stimulation in BMDM. Rothlin and colleagues^18^ reported that TAM activation upon TLR stimulation in dendritic cells usurps the type 1 interferon receptor (IFNAR)-STAT1 cassette to induce TLR suppressors, suppressor of cytokine signaling 1 (SOCS1) and SOCS3. Here, we demonstrated that Gas6 rapidly enhanced STAT1 phosphorylation at Tyr-701 in BMDM in the absence of TLR signaling. Gas6-induced STAT1 phosphorylation was also shown in dendritic cells with a similar time course^18^. However, the LXR agonist T0901317 did not induce STAT1 phosphorylation, whereas it enhanced *LXRα* and *LXRβ* mRNA expression in BMDM. These data suggest that not only LXR ligands, but also extracellular signals, contribute to LXR expression and activity in macrophages. Importantly, the inhibition of STAT1 signaling via genetic ablation or pretreatment with the JAK2 inhibitor tyrphostin AG490 or the STAT1 inhibitor fludarabine resulted in the reduction of enhanced LXRα and LXRβ mRNA and/or protein abundance by Gas6 treatment in BMDM. The induction of LXR target genes, including *ABCA1, ABCG1, ApoE, AIM, Arg2*, and *VEGF* was also markedly reduced in *STAT1*-deficient BMDM. However, the abundance of STAT1 mRNA and protein in BMDM was not affected during the 24 h after Gas6 stimulation (data not shown). Moreover, fludarabine prevented the enhancement of transcriptional activity from the LXRE promoter by Gas6 in RAW 264.7 cells. Collectively, these data strongly suggest that STAT1 phosphorylation downstream of Gas6/Mer leads to LXRα and LXRβ expression and activation in mouse BMDM and extensively mediates the LXR transcriptional response. In contrast, Pascual-García and colleagues demonstrated that STAT1 activation by IFN-γ in mouse BMDM negatively regulates the induction of selective LXR target genes, including *ABCA1* and *sterol response element binding protein 1c*^24^. Moreover, in their hand, IFN-γ did not alter the expression of LXR isoforms. In comparison with our findings, this STAT1 dichotomy may be in part explained by alternative posttranslational modifications of LXR, or alternative recruitment of cofactors^18^,^24^,^25^ in different activating systems of Gas6/Mer and IFN-γ/its receptor.

In addition to STAT1 activation, STAT3 phosphorylation was shown under Gas6 stimulation in BMDC from WT but not *Mer*^−/−^ mice, indicating that STAT3 is activated in a Gas6/Mer-dependent manner^19^. However, we found that STAT3 phosphorylation was not induced in BMDM treated with Gas6, suggesting that STAT3 is not involved in Gas6/Mer signaling-mediated LXR induction and activation. In their study, Gas6 also induced STAT1 phosphorylation in BMDC^19^. However, their findings suggested that STAT1 signaling does not contribute to the Gas6/Mer-induced inhibition of BMDC. In our studies, we found that Gas6 enhanced STAT1 and Akt phosphorylation in BMDC from both WT and Mer^−/−^ mice, indicating that the enhanced STAT1 and Akt activation is Mer independent. Unlike to BMDM, Gas6 did not induce LXRα and LXRβ gene expression in BMDC. These results demonstrate that although phosphorylation of Akt and STAT1 was detected in Mer^+/+^ BMDC treated with Gas6, Gas6-Mer-PI3K/Akt-STAT1-LXR pathway appears to be macrophage specific. Therefore, the requirement for STAT1 signaling in macrophages, but not dendritic cells, is not astonishing because Gas6/Mer signaling regulates distinct STAT pathways in these 2 types of antigen-presenting cells^19^,^26^.

Recent reports have demonstrated that the Mer pathway involving PI3K/Akt and nuclear factor (NF)-κB in macrophages is responsible for the down-modulation of proinflammatory signals without dependence on new protein synthesis^21^,^27^. Our previous study demonstrated that the Akt-specific inhibitor MK2206 reduces Gas6-induced LXR mRNA and protein expression in RAW 264.7 cells^16^. Here, we confirmed that the PI3K/Akt pathway links Gas6/Mer signaling to LXR expression and its functional activity in mouse BMDM, as PI3K inhibitors such as wortmannin and LY294002 or the Akt inhibitor MK2206 markedly reduced Gas6-induced LXR mRNA and protein expression in BMDM. In addition, MK2206 reduced Gas6-induced protein abundances of Arg2 and VEGF. Notably, a similar time course of Akt phosphorylation with STAT1 was shown in BMDM upon stimulation with Gas6, but the peak response of Akt phosphorylation occurred earlier, compared with that of STAT1 phosphorylation (15 min vs. 30 min). These time course data imply that the PI3K/Akt pathway may mediate Gas6-induced STAT1 phosphorylation. Indeed, inhibitors of PI3K or Akt apparently blocked STAT1 phosphorylation by Gas6. Similarly, it has been reported that PI3K mediates TLR4-induced STAT1 tyrosine phosphorylation in RAW 264.7 macrophages^28^. Additionally, Akt phosphorylation was markedly reduced in BMDM treated with fludarabine and in *STAT1*-deficient BMDM. Taken together, these data reveal a positive cross-talk between the PI3K/Akt and STAT1 pathways involving Gas6-induced enhancement of LXR expression and activation in BMDM. A positive cross-talk between serine phosphorylation of STAT1 and PI3K signaling in HIV-1-induced blood-brain barrier (BBB) dysfunction has been reported^29^. However, we found that Gas6 did not induce serine phosphorylation of STAT1 in BMDM. Phosphorylation of Tyr-701 alone is sufficient to generate STAT multimers that possess DNA binding activity^30^, although phosphorylation of Ser-727 is required for maximal transcriptional activity of STAT1^31^. Future studies of other candidate molecules, such as all three MAP kinases, including p38, ERK, JNK, and Src, or the integral pathway between PI3K/Akt and STAT1 that links Mer to LXR expression and functional activity are essential.

Through transcriptional profiling, *Arg2* was identified as a direct target of LXR/RXR heterodimers^20^. Induction of *Arg2* was more prominent in cells expressing LXRα as compared with those expressing LXRβ. In the present study, we also found that LXRα siRNA more significantly inhibited Gas6-induced *Arg2* mRNA expression than LXRβ siRNA in RAW 264.7 cells. Thus, we focused the role of LXRα on the transcriptional regulation of the *Arg2* gene. Mutation of the LXRE or STAT1 binding site in the *Arg2* promoter completely blocked Gas6-induced promoter activity, indicating the LXRE and STAT1 binding sites are necessary for Gas6-induced *Arg2* transcription. Next, we confirmed the physical binding of LXRα and STAT1 to the *Arg2* promoter in BMDM using ChIP analyses, which was abolished in *STAT1*-deficient BMDM. These ChIP data support the idea that these two transcription factors are likely to be in the same DNA binding complex *in vivo* and functionally interact with each other on the *Arg2* promoter. Previous studies in macrophages showed that physical and functional interaction of STAT1 and another nuclear transcription factor, RXRα, leads to apolipoprotein C-II (*Apoc2*) gene transactivation^32^.

The promoter of LXRα contains LXREs, enabling an autoregulatory loop in adipocytes and macrophages^33^. This may be important for the induction of LXR above a threshold necessary to regulate certain target genes^34^. Several transcription factors, such as the nuclear factor 1 (NF1), CCAAT/enhancer-binding proteins (C/EBPs), and glucocorticoid receptors (GRs) were shown to affect the transcriptional potential of the mouse LXRα promoter^35^,^36^,^37^. Further investigation is required to elucidate details of the direct and indirect mechanisms for STAT1-dependent induction of LXRα and LXRβ genes in macrophages.

Arg2 is most highly expressed in kidney, prostate, and immune cells such as monocytes/macrophages^38^. An imbalance in the iNOS/Arg2 ratio has been postulated to play a crucial role in regulating the immune response and macrophage activation. Since both Arg2 and iNOS utilize arginine as a common substrate, induction of Arg2 in macrophages would be expected to inhibit NO production^39^,^40^ and are termed anti-inflammatory macrophages or alternative (M2) activated macrophages^41^,^42^,^43^,^44^,^45^,^46^. Forced expression of Arg2 mimics the inhibitory effect of LXR activation on macrophage NO production^9^,^20^. In our system, we found that inhibitory effect of Gas6 on LPS-induced nitrite production was reversed after silencing the LXR isoforms individually in BMDM. Importantly, these inhibitory effects of Gas6 were not shown in BMDM pretreated with the STAT1 inhibitor or transfected with *STAT1* siRNA. Gas6-induced reduction of NO production was not a consequence of decreased iNOS, but rather increased Arg2 protein expression, as iNOS protein abundance was not affected but Arg2 protein was enhanced by Gas6 treatment in LPS-stimulated BMDM. These results suggest that Gas6/Mer signaling uses the STAT1 pathway to promote LXR participation in nitrogen metabolism in macrophages, controlling excess NO production during inflammatory responses through the transcriptional activation of *Arg2*.

Alciato and colleagues demonstrated that Gas6/Mer pathway involving PI3K/Akt and nuclear factor (NF)-κB inhibits TNF-α, IL-1, and IL-6 secretion by LPS-stimulated U937 macrophages^21^, indicating modulation of macrophage functional polarity from M1 toward an anti-inflammatory M2 phenotype^47^. Zizzo and colleagues showed that Gas6 significantly enhances LPS-induced IL-10 production via Mer in M-CSF-differentiated human M2 macrophages^22^. On the other hand, our previous *in vivo* studies demonstrated that Gas6-Mer signaling-dependent upregulation of LXRα and LXRβ leads to the expression of pro-resolving cytokines, such as TGF-β and hepatocyte growth factor (HGF) for tissue resolution of acute inflammation^16^. In the present study, we also provide more evidence to support M1/M2 conversion since Gas6 treatment enhanced IL-10 secretion in LPS-stimulated BMDM. This effect was prevented by pharmacological inhibition of Akt or knockdown of LXRα protein with LXRα-specific siRNA. However, STAT1-specific siRNA did not significantly inhibit the Gas6-induced increase in IL-10 production. These data suggest that the Akt-LXR pathway, excluding STAT1, is involved in Gas6-induced increase in IL-10 production in BMDM upon stimulation with LPS.

In addition, Gas6 seems also to modulate macrophage migration. Gas6 treatment directly inhibits the chemotactic migration of BMDM toward chemoattractants. Interestingly, pretreatment with fludarabine or MK2206 reversed this suppressive effect of Gas6 on chemotaxis. Similarly, knockdown of LXRα protein by transfection of BMDM with a LXRα-specific siRNA reversed the Gas6-induced inhibition of chemotaxis. These data suggest that Gas/Mer signal-induced Akt-STAT1-LXR pathway is involved in modulating macrophage function with low chemotaxis. Taken together, the post-receptor signaling pathway between Mer and LXR expression might include Akt and STAT1 coordinately or individually, switching to antiinflammatoy M2 phenotype. Nonetheless, future studies are needed to fully understand potential candidate molecules that mediate Gas6-induced modulating effect on macrophage functional polarization from M1 toward an anti-inflammatory M2 phenotype.

In our previous work, we demonstrated the Mer anti-inflammatory pathway *in vivo* in acute lung injury^17^. Pretreatment with anti-Mer neutralizing antibody significantly reduced LPS-induced Mer activation, Akt phosphorylation, and STAT1 activation in lung tissue. In parallel, LPS-induced TLR4-proximal signaling (NF-κB signaling), proinflammatory mediator production, inflammatory cell accumulation and destruction of blood-pulmonary epithelial cell barrier integrity were augmented by anti-Mer antibody. In the present study, we further demonstrated using this *in vivo* model that anti- Mer antibody treatment results in decreases in mRNA and protein abundances of LXRα and Arg2 in lung tissue and concomitant increases in NO production in BAL fluid at 24 h after LPS treatment. Our present *in vivo* results suggest the possibility that Gas6/Mer signaling-induced LXRα/Arg2 pathway during LPS-induced acute lung injury leads to downregulated NO production, which in part contributes to the resolution of acute lung injury. Despite advances in understanding the mechanisms of Mer-dependent modulation of the innate immune response, further studies are needed to understand fully the role of Gas6/Mer/Akt/STAT1/LXR pathway for resolving inflammatory response in acute lung injury.

Our findings suggest that Gas6/Mer signaling enhances LXR mRNA and protein abundance and activity in mouse BMDM, leading to induction of target genes involved in lipid metabolism as well as the anti-inflammatory and resolving response. Here, we have elucidated a novel role of STAT1 and a positive cross-talk with the PI3K/Akt pathway, which connects Mer signaling to increases in LXR expression and activation. Moreover, STAT1 might facilitate LXRα activity on the *Arg2* gene promoter or itself act as a coactivator to provide more efficient transactivation. Interaction of STAT1 and LXR may control excess NO production during inflammatory responses via enhanced *Arg2* induction. We provide more evidence that GAS6/Mer signaling induces functional switch in BMDM toward a pro-resolution M2 phenotype with blocked chemotaxis and increased IL-10 secretion via LXR pathway. In addition, our previous and present *in vivo* data implicate that Gas6/Mer/Akt/STAT1 signaling modulates NO production via activation of LXR/Arg2 pathway during LPS-induced acute lung injury, which in part promotes the resolution of acute lung injury^17^.

## Materials and Methods

### Reagents

Recombinant mouse Gas6 was purchased from R&D Systems (Minneapolis, MN, USA). Wortmanin and LY294002 were purchased from Sigma-Aldrich (St. Louis, MO, USA). MK220 and T0901317 were obtained from Cayman Chemical (Ann Arbor, MI, USA). The Gene-Specific Relative RT-PCR kit was obtained from Invitrogen (Carlsbad, CA, USA). M-MLV reverse transcriptase was from Enzynomics (Hanam, Korea). The following antibodies were used: anti-LXRβ, anti-ABCG1, anti-Aim, anti-Arg2, and anti-VEGF (all from Santa Cruz Biotechnology, Santa Cruz, CA, USA); anti-LXRα and anti-ApoE (Abcam, Cambridge, UK); anti-ABCA1 (Novus Biologicals, Littleton CO, USA); anti-phospho-STAT1 (Tyr-701, Ser-727) and total STAT1 (Cell Signaling Technology, Beverly, MA, USA) and anti-α-tubulin (Sigma-Aldrich). DNA polymerase Klenow fragment and dNTPs were obtained from Intron Biotechnology (Seoul, Korea).

### Mice

Pathogen-free, male C57BL/6 mice, 6 to 8 weeks old, weighing 19 to 21 g were purchased from Orient Bio (Sungnam, Korea). B6.129-*Mertk*^*tm1Gr1*^/J (*MerTK*^−/−^) and wild-type (WT) control mice with an identical background (B6.129SF2/J) were obtained from The Jackson Laboratory (Bar Harbor, Maine, USA). *STAT1*^−/−^ mice were from the Charles River Laboratory. *STAT1*^−/−^ mice were on a C57BL/6 background (Orient Bio). *MerTK*^−/−^, *STAT1*^−/−^, and the respective WT mice were age-matched (7 to 10 weeks old) and sex-matched for all experiments. The Animal Care Committee of the Ewha Medical Research Institute approved the experimental protocol. Mice were cared for and handled in accordance with the National Institutes of Health (NIH) Guide for the Care and Use of Laboratory Animals.

### Cells

Primary BMDM were prepared from C57BL/6 mice, *MerTK*^−/−^ mice, *STAT1*^−/−^ mice, and WT mice as described previously^48^. Briefly, BMDM were differentiated from murine bone marrow myeloid stem cells. Bone marrow cells were cultured for 7–10 days with DMEM supplemented with 10% L929 supernatant containing 10% heat-inactivated fetal bovine serum (FBS), 1 mM sodium pyruvate, 50 U/ml penicillin, 50 μg/ml streptomycin, and 5 × 10^−5^ M 2-mercaptoethanol. BMDM differentiation was confirmed by FACS analysis using anti-CD11b. Murine RAW 264.7 macrophages [American Type Culture Collection (ATCC), Manassas, VA, USA] were plated at a density of 10^6^ cells/ml and incubated overnight in Dulbecco’s modified Eagle’s medium (DMEM, Media Tech Inc., Washington, DC, USA) supplemented with 10% FBS, 2 mM L-glutamine, 100 U/ml penicillin, and 100 μg/ml streptomycin at 37 °C and 5% CO_2_. Prior to stimulation, the medium was replaced with serum-free X-VIVO medium (Lonza, Basel, Switzerland). On the other hand, BMDC were induced to differentiate by the addition of recombinant mouse GM-CSF (rmGM-CSF, R&D Systems) (20 ng/ml) every 3 days and LPS (1 μl/ml, Sigma-Aldrich) on day 10. After another 48 h, cells and medium were collected for further assays.

### Quantitative real-time PCR (qPCR)

Gene expression was analyzed using real-time qPCR on a StepOnePlus system (Applied Biosystems, Foster City, CA, USA). For each qPCR assay, a total of 50 ng of cDNA was used. Primer sets (Supplementary Table 1) for PCR amplifications were designed using Primer Express software (Applied Biosystems). The abundances of the cDNAs of interest were normalized to that of hypoxanthine guanine phosphoribosyl transferase (*Hprt)* cDNA^49^ and are reported as the fold-change in abundance compared to the appropriate controls.

### Immunoblotting analysis

Mouse BMDM and RAW 264.7 macrophages (10^6^ cells/ml) were plated and incubated in serum-free medium overnight at 37 °C in 5% CO_2_. The stimulated cells were lysed in 0.5% Triton X-100 lysis buffer and resolved on a 10% SDS-PAGE gel. Separated proteins were electrophoretically transferred onto a nitrocellulose membrane. Standard Western blotting techniques were then applied.

### Transient cell transfection with siRNAs

BMDM or RAW 264.7 cells were transiently transfected with siRNA targeting *STAT1* (75 nM), *LXRα* (100 nM), *LXRβ* (100 nM), or control siRNA (Bioneer, Seoul, Korea) using 5 μl of siRNA transfection reagent (Genlantis, San Diego, CA) according to the manufacturer’s protocol. The sequences used for *STAT1* knockdown were sense 5′-CACAGUUUUAUCCUGAUGA-3′ and antisense 5′-UCAUCAGGAUAAAACUGUG-3′. The sequences used for *LXRα* knockdown were sense 5′-CGUAGCAUUAAGGGAGAGU-3′ and antisense 5′-ACUCUCCCUUAAUGCUACG-3′. The sequences used for *LXRβ* knockdown were sense 5′-ACGCUUACACCUCAGCCUA-3′ and antisense 5′-UAGGCUGAGGUGUGUAAGCGU-3′. The sequences for the control siRNA were sense 5′-CCUACGCCACCAAUUUCGU-3′ and antisense 5′-ACGAAAUUGGUGGCGUAGG-3′. The cells were then incubated in serum-free culture medium for 48 or 66 h.

### Luciferase reporter assay

The LXRE-luciferase vector (TK-LXREx3-Luc vector) was a kind gift from Dr. D. J. Mangelsdorf (University of Texas Southwestern Medical Center). The mouse *Arg2* promoter (−1,840 to +245; 2085 bp) was obtained by PCR using mouse genomic DNA as a template and inserted into the luciferase reporter plasmid (pGL3-basic; Promega, Madison, WI). The LXRE (TGGCCTCTAGTAACCA) and STAT1 (TTCCCGGGAA) sites on the *Arg2* promoter were mutated to TGGAATCTAGTAATTA and GGTCCGGTTT, respectively (mutated nucleotides are underlined). RAW 294.7 cells were co-transfected with promoter reporters and Renilla luciferase vector (phRL-TK) using X-tremeGENE 9 DNA Transfection Reagent (Roche Applied Science, Penzberg, Upper Bavaria, Germany). After 24 h, firefly and Renilla luciferase activity was measured using a dual-luciferase reporter assay system (Promega) on the Synergy HT Multi-Mode Microplate Reader (BioTek Instruments, Winooski, VT).

### Chromatin immunoprecipitation assay

BMDM cells were cross-linked with 1% formaldehyde and chromatin was isolated and sonicated using a Fisher Dismembrator Model 100 (20 s pulse for 8 cycles)^50^. Fragmented chromatin was immunoprecipitated by LXRα (Abcam, ab41902) and STAT1 (Cell Signaling Technologies, #9172) antibodies. Immunoprecipitated DNA samples were analyzed by real-time qPCR of target regions. PCR primers and TaqMan probe sequences are listed in Supplementary Table 2.

### Chemotaxis assay

Chemotaxis assays were performed using Transwell chambers (Corning Inc, Corning, NY (6.5 mm diameter insert, 8.0 μm pore size, polycarbonate membrane) as described^51^. In brief, BMDM (5 × 10^5^ cells/well) in serum-free DMEM/F12 containing Gas6 (400 ng/ml) with or without fludarabine (1 μM) or MK2206 (1 μM) were seeded in the upper chambers and the lower chambers were filled with DMEM/F12 supplemented with 10% FBS at 37 °C for 18 h. After fixation in 4% paraformaldehyde, the nonmigrated cells on the upper surface of the membrane were scraped off with a cotton swap. The cells on the lower surface were stained using 0.05% crystal violet, and washed with distilled water. Three random microscopic fields (10X magnification) were photographed and counted per chamber, and results were expressed as the mean ± SEM of the cells on the bottom from the replicate wells.

### Animal protocols

Specific pathogen-free male BALB/C mice (Orient Bio, Sungnam, Korea) weighing 19–21 g were used in all experiments. The Animal Care Committee of the Ewha Medical Research Institute approved the experimental protocol. Mice were cared for and handled in accordance with the National Institutes of Health (NIH) Guide for the Care and Use of Laboratory Animals. Mouse pharyngeal aspiration was used to administer a test solution (30 μl) containing LPS (1.5 mg/kg)^17^. Control mice were administrated sterile saline (0.9% NaCl). Animals were intravenously given 2.0 mg/kg polyclonal goat anti-mouse Mer antibody (AF591, R&D Systems)^52^,^53^, or control goat IgG antibody (R&D Systems) 1 h before LPS treatment and sacrificed 24 h post-LPS.

### BAL fluid and lung tissue

BAL was performed through a tracheal cannula using 0.7 ml aliquots of ice-cold Ca^2+^/Mg^2+^-free phosphate-buffered medium (145 mM NaCl, 5 mM KCl, 1.9 mM NaH_2_PO_4_, 9.35 mM Na_2_HPO_4_, and 5.5 mM dextrose; pH 7.4) to a total of 3.5 ml for each mouse. After BAL, BAL fluid and lungs were r immediately frozen in liquid nitrogen, and stored at −70 °C.

### Measurement of nitrite levels

Mouse BMDM were transfected with control vehicle, *LXRα* siRNA, or *STAT1* siRNA for 18 hours and then stimulated with Gas6 for 24 h before LPS treatment. Accumulated nitrite was measured in the cell supernatant or BAL fluid by Griess reagent.

### Statistical analysis

Data are expressed as mean ± SEM. Analysis of variance (ANOVA) was applied for multiple comparisons and Tukey’s post-hoc test was applied where appropriate. The Student’s *t* test was used for comparisons of two sample means. A *p*-value less than 0.05 was considered statistically significant.

## Additional Information

**How to cite this article**: Kim, S.-Y. *et al*. Liver X receptor and STAT1 cooperate downstream of Gas6/Mer to induce anti-inflammatory arginase 2 expression in macrophages. *Sci. Rep.* **6**, 29673; doi: 10.1038/srep29673 (2016).

## Supplementary Material

Supplementary Information
