# Supplementary material for: Liver X receptor and STAT1 cooperate downstream of Gas6/Mer to induce anti-inflammatory arginase 2 expression in macrophages
Source: Sci Rep. 2016 Jul 13;6:29673. doi: 10.1038/srep29673 (PMC4942780; doi:10.1038/srep29673)
Supplement: Supplementary Information [file srep29673-s1.pdf]

Liver X receptor and STAT1 cooperate downstream of Gas6/Mer to induce anti-inflammatory arginase 2 expression in macrophages

Si-Yoon Kim[1,2*], Eun-Jin Lim[2*], Young-Ho Ahn[2,4], Eun-Mi Park[2,3], Hee-Sun Kim[2,4], Jihee Lee Kang,[1,2†]

[1]Department of Physiology, School of Medicine, Ewha Womans University, Seoul 158-710, Korea

[2]Tissue Injury Defense Research Center, School of Medicine, Ewha Womans University, Seoul 158-710, Korea

[3]Department of Pharmacology, School of Medicine, Ewha Womans University, Seoul 158-710, Korea

[4]Department of Molecular medicine, School of Medicine, Ewha Womans University, Seoul 158-710, Korea

*These authors contributed equally to this work.

[†]Corresponding author: Dr. Jihee Lee Kang, Department of Physiology, Tissue Injury Defense Research Center, School of Medicine, Ewha Womans University, 911-1 Mok-6-dong, Yangcheon-ku, Seoul 158-056, Korea (Telephone) 82-2-2650-5719, (Fax) 82-2-2650-5717. E-mail: jihee@ewha.ac.kr

Supplementary Table S1. Sequences of primers used for real-time PCR analysis.

Target gene (Mouse)	primer	Sequences (5' to 3')
Nuclear Receptor subfamily 1, Group H, Member 3 (NR1H3, LXRα)	forward	AGGAGTGTCGACTTCGCAAA
	reverse	CTCTTCTTGCCGCTTCAGTTT
Nuclear Receptor subfamily 1, Group H, Member 2 (NR1H2, LXRβ)	forward	GCTCTGCCTACATCGTGGTC
	reverse	CTCATGGCCCAGCATCTT
RXR	forward	CAAACATGGGGCTGAACC
	reverse	GCCCACTCCACAAGAGTGA
ATP-binding cassette, sub-family A, member 1 (ABCA1)	forward	CGCAAGCATATGCCTCAT
	reverse	CCCATTACATAACACATGGCT
ATP-binding cassette, sub-family G, member 1 (ABCG1)	forward	GCTACATCATGCAGGACGAC
	reverse	CGTCTGCCTTCATCCTTCTC
Apolipoprotein E (ApoE)	forward	AGGATCTACGCAACCGACT
	reverse	CTGCCTTGTACACAGCTA
Apoptosis inhibitor of macrophage (AIM)	forward	GAGGACACATGGATGGAATGT
	reverse	ACCCTTGTGTAGCACCTCCA
Arginase2 (Arg2)	forward	GTGTCACCATGGGAGGAGA
	reverse	CATGAGCATCAACCCAGATG
Vascular endothelial growth factor A (VEGF-A)	forward	GCAGCTTGAGTTAAACGAACG
	reverse	GGTTCCCGAAACCCTGAG
Arginase1 (Arg1)	forward	GTGGGGAAAGCCAATGAAG
	reverse	GCTTCCAACTGCCAGACTGT
YM1	forward	TCTGGGTACAAGATCCCTGAA
	reverse	TCATATGGAGATTTATAGAGGGGACT
Hypoxanthine guanine phosphoribosyl transferase (Hprt)	forward	CAGACTGAAGAGCTACTGTAATG
	reverse	CCAGTGTCAATTATATCTTCAAC

Supplementary Table S2. PCR primers and TaqMan probes

Arginase 2 promoter	primer	Sequences (5' to 3')
**LXRE site**	forward	TGGATTTGTTTCAGCCTCCT
	reverse	CTCTTAACTGTTCAGCTACCTCTC
	TaqMan	FAM-AGTTGGCCTCTAGTAACCAGTGCTCT-ZEN-BHQ1
**STAT1 site**	forward	GTTCCACTGAGGTCTCCAAC
	reverse	ACCTGTCCTCTCTTTCCCT
	TaqMan	FAM-TTATGGTATCCGCCTTGGTGGCTT-ZEN-BHQ1
**Negative control**	forward	AAGGTGTAGCCCGGATTAAAG
	reverse	CTTGAGCGGTAGTGGCTATG
	TaqMan	FAM-ATCCCAGATGAAAGGCATAGCCCA-ZEN-BHQ

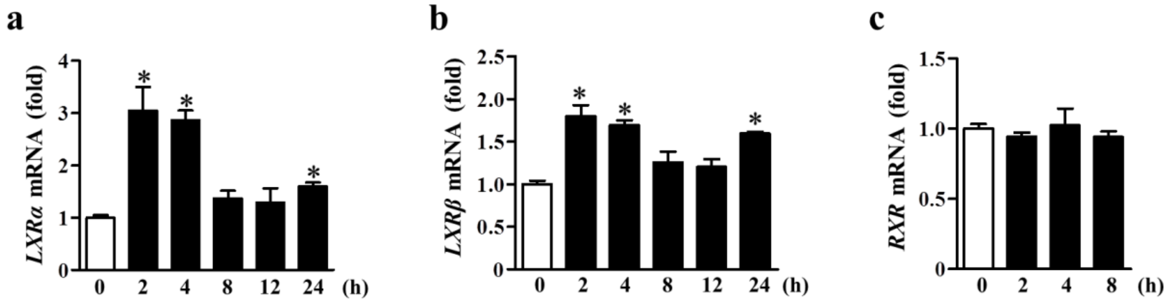

Supplementary Fig. S1. Expression of LXRα and LXRβ is enhanced by Gas6 treatment in BMDM. Mouse BMDM were stimulated with 400 ng/ml Gas6 for the indicated times. The amounts of the *LXR*α, *LXR*β, and *RXR* mRNAs were analyzed by real-time PCR and normalized to that of *Hprt* mRNA. Data in all bar graphs are means ± SEM of three independent experiments. **P* < 0.05 compared with control.

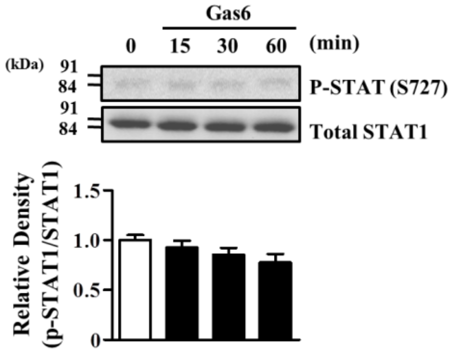

Supplementary Fig. S2. Gas6 treatment does not induce serine phosphorylation in STAT1 in BMDM. Mouse BMDM were stimulated with 400 ng/ml Gas6 for the indicated times. The relative abundances of total STAT1 and serine phosphorylated STAT1 (S727) were determined by Western blotting analysis. Bar graphs show densitometric analysis of the relative abundances of serine phosphorylated STAT1 (S727) over time. Data in all bar graphs are means ± SEM of three independent experiments.

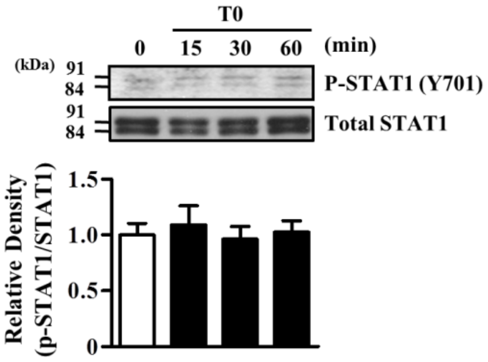

Supplementary Fig. S3. T0901317 does not induce tyrosine phosphorylation of STAT1 in BMDM. Mouse BMDM were stimulated with 1 μM T0901317 for the indicated times. The relative abundances of total STAT1, and tyrosine phosphorylated STAT1 (Y701) were determined by Western blotting analysis. Bar graphs show densitometric analysis of the relative abundances of tyrosine phosphorylated STAT1 (Y701) over time. Data in all bar graphs are means ± SEM of three independent experiments.

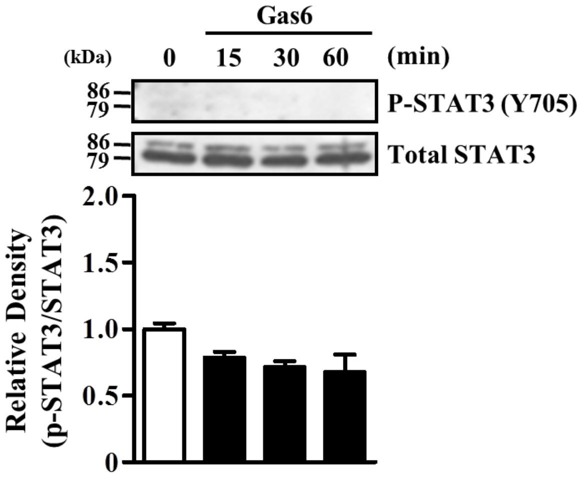

Supplementary Fig. S4. Gas6 treatment does not induce phosphorylation of STAT3 in BMDM. Mouse BMDM were stimulated with 400 ng/ml Gas6 for the indicated times. The relative abundances of total STAT3 and phosphorylated STAT3 (Y705) were determined by Western blotting analysis. Bar graphs show densitometric analysis of the relative abundances of serine phosphorylated STAT3 (Y705) over time. Data in all bar graphs are means ± SEM of three independent experiments.

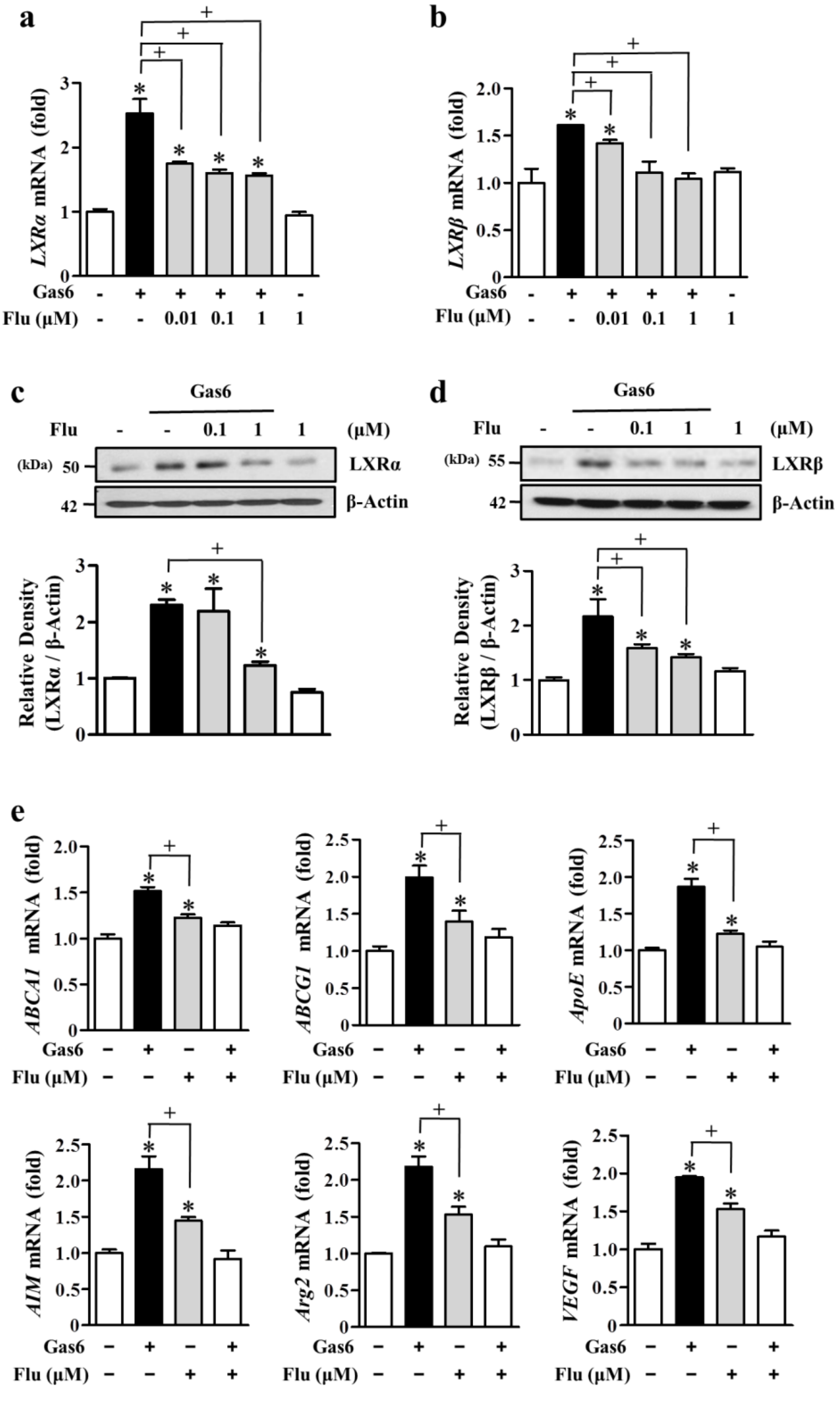

Supplementary Fig. S5. The STAT1 specific inhibitor fludarabine inhibits Gas6-induced

increases in LXRα, LXRβ, ABCA1, ABCG1, ApoE, AIM, Arg2, and VEGF expression in BMDM. Mouse BMDM were pretreated with vehicle or the STAT1-specific inhibitor fludarabine at the indicated concentrations (a-d) or 1μM (e) before 400 ng/ml Gas6 treatment. At 4 h after Gas6 treatment, the amounts of the *LXRα and LXRβ* mRNAs (a,b) and 24 h after Gas6 treatment, the amounts of *ABCA1, ABCG1, ApoE, AIM, Arg2,* and *VEGF* mRNA (e) were analyzed by real-time PCR and normalized to that of *Hprt* mRNA. (c,d) At 18 h after Gas6 treatment, samples were analyzed by Western blotting to determine the relative abundances of LXRα and LXRβ proteins. β-actin was used as a loading control. Bar graphs show the densitometric analysis of the relative abundances of the indicated proteins. Data in all bar graphs are means ± SEM of three independent experiments. *$P < 0.05$ compared with control; $^{+}P < 0.05$ as indicated.

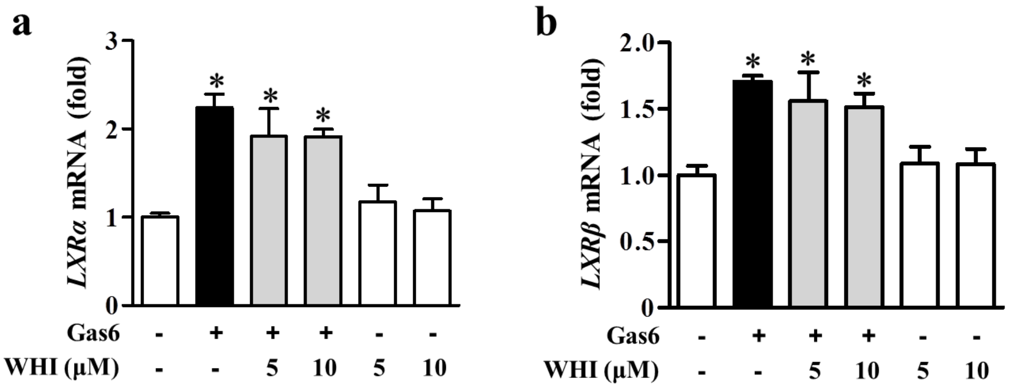

Supplementary Fig. S6. The JAK3 inhibitor does not affect Gas6-induced *LXRα* and *LXRβ* mRNA expression in mouse BMDM. Mouse BMDM were pretreated with vehicle or the indicated concentrations of the JAK3 inhibitor WHI-P131 before 400 ng/ml Gas6 treatment. The amounts of the *LXRα* and *LXRβ* mRNAs were analyzed by real-time PCR and normalized to that of *Hprt* mRNA. Data in all bar graphs are means ± SEM of three independent experiments.

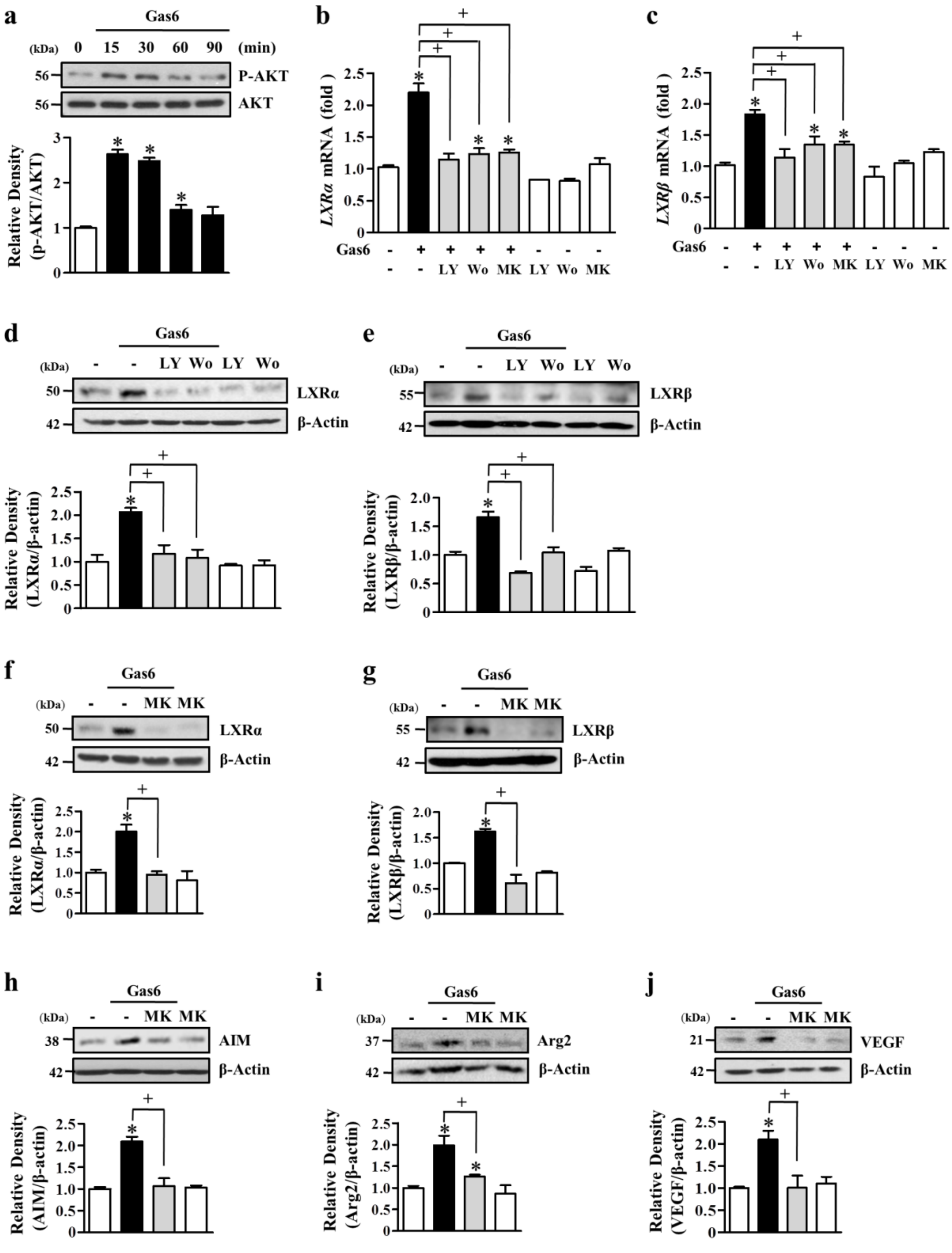

Supplementary Fig. S7. Inhibition of PI3K/Akt pathway reduces Gas6-induced LXRα and LXRβ mRNA and protein expression in BMDM. (a) Mouse BMDM were stimulated with 400 ng/ml Gas6 for the indicated times. The relative abundances of total and

phosphorylated Akt proteins were determined by Western blotting analysis. Bar graphs show densitometric analysis of the phosphorylated Akt protein abundances, normalized to that of total protein. (b-j) Mouse BMDM were pretreated with vehicle or the PI3K-specific inhibitor LY294002 (10 μM), wortmanin (100 nM), or the Akt inhibitor MK2206 (1 μM) before 400 ng/ml Gas6 treatment. (b,c) At 4 h after Gas6 treatment, the amounts of the *LXRα and LXRβ* mRNAs were analyzed by real-time PCR and normalized to that of *Hprt* mRNA. At 18 h (d-g) or 24 h (h-j) after Gas6 treatment, samples were analyzed by Western blotting to determine the relative abundances of LXRα, LXRβ, AIM, Arg2, and VEGF proteins. β-actin was used as a loading control. Bar graphs show the densitometric analysis of the relative abundances of the indicated proteins. Data in all bar graphs are means ± SEM of three independent experiments. $^{*}P < 0.05$ compared with control; $^{+}P < 0.05$ as indicated.

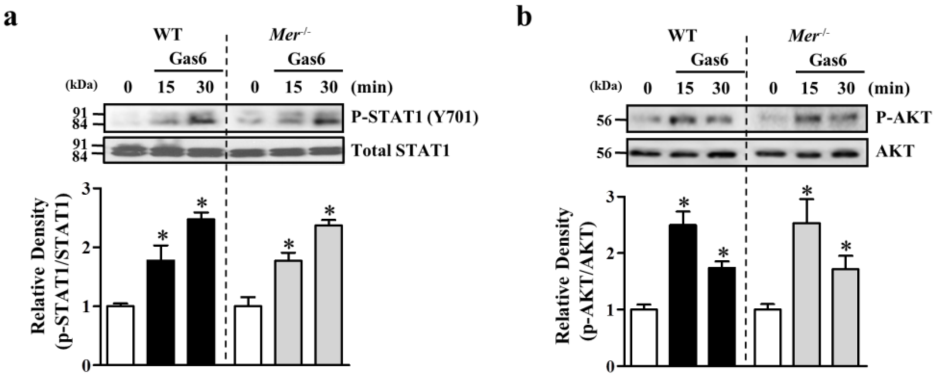

Supplementary Fig. S8. Gas6-induced phosphorylation of STAT1 and Akt is Mer independent in BMDC. Mouse BMDC from wild-type or *Mer*$^{-/-}$ mice were stimulated with 400 ng/ml Gas6 for the indicated times. (a,b) The relative abundances of total STAT1, phosphorylated STAT1 (Y701), total Akt, and phosphorylated Akt proteins were determined by Western blotting analysis. The relative densitometric intensity was determined for each band and normalized to the indicated proteins. Data in all bar graphs are means ± SEM of three independent experiments. *$P < 0.05$ compared with control.

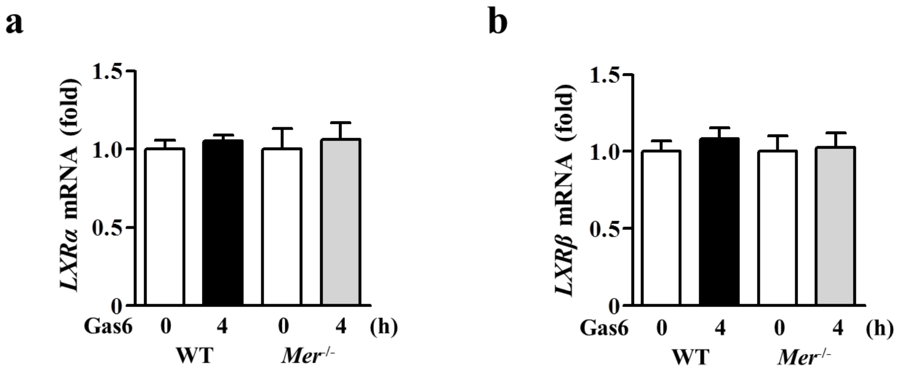

Supplementary Fig. S9. Gas6 does not enhance LXRα and LXRβ gene expression in BMDC. Mouse BMDC were stimulated with 400 ng/ml Gas6 for 4 h. The amounts of the *LXRα* or *LXRβ* mRNAs were analyzed by real-time PCR and normalized to that of *Hprt* mRNA. Data in all bar graphs are means ± SEM of three independent experiments. $^*P < 0.05$ compared with control.

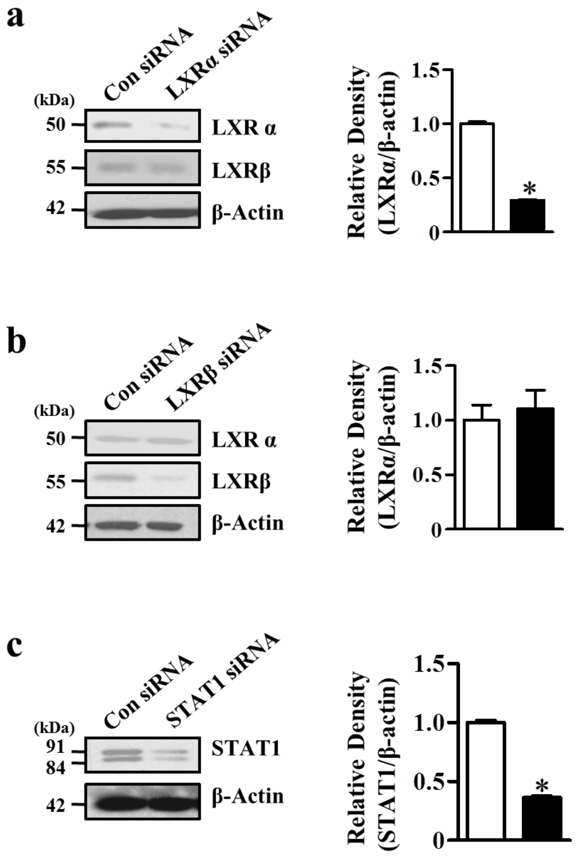

Supplementary Fig. S10. LXRα, LXRβ, or STAT1 expression is reduced after transfection of BMDM with its specific siRNA. Mouse BMDM were transfected with either control siRNA, LXRα, LXRβ or STAT1-specific siRNA. After 66 h the relative abundances of LXRα, LXRβ, and STAT1 proteins were determined by Western blotting analysis. The relative densitometric intensity was determined for each band and normalized to β-actin. Data in all bar graphs are means ± SEM of three independent experiments. $*P < 0.05$ compared with control.
